# Regulation of longevity by depolarization-induced activation of PLC-β–IP_3_R signaling in neurons

**DOI:** 10.1073/pnas.2004253118

**Published:** 2021-04-15

**Authors:** Ching-On Wong, Nicholas E. Karagas, Jewon Jung, Qiaochu Wang, Morgan A. Rousseau, Yufang Chao, Ryan Insolera, Pushpanjali Soppina, Catherine A. Collins, Yong Zhou, John F. Hancock, Michael X. Zhu, Kartik Venkatachalam

**Affiliations:** ^a^Department of Integrative Biology and Pharmacology, McGovern Medical School at the University of Texas Health Sciences Center, Houston, TX 77030;; ^b^Department of Biological Sciences, Rutgers University, Newark, NJ 07102;; ^c^Graduate Program in Biochemistry and Cell Biology, MD Anderson Cancer Center and UTHealth Graduate School of Biomedical Sciences, Houston, TX 77030;; ^d^Department of Molecular, Cellular, and Developmental Biology, University of Michigan, Ann Arbor, MI 48109

**Keywords:** longevity, aging, ER Ca^2+^ signaling, lysosomes, neuronal excitability

## Abstract

Mitochondrial ATP production is a well-known regulator of neuronal excitability. The reciprocal influence of plasma-membrane potential on ATP production, however, remains poorly understood. Here, we describe a mechanism by which depolarized neurons elevate the somatic ATP/ADP ratio in *Drosophila* glutamatergic neurons. We show that depolarization increased phospholipase-Cβ (PLC-β) activity by promoting the association of the enzyme with its phosphoinositide substrate. Augmented PLC-β activity led to greater release of endoplasmic reticulum Ca^2+^ via the inositol trisphosphate receptor (IP_3_R), increased mitochondrial Ca^2+^ uptake, and promoted ATP synthesis. Perturbations that decoupled membrane potential from this mode of ATP synthesis led to untrammeled PLC-β–IP_3_R activation and a dramatic shortening of *Drosophila* lifespan. Upon investigating the underlying mechanisms, we found that increased sequestration of Ca^2+^ into endolysosomes was an intermediary in the regulation of lifespan by IP_3_Rs. Manipulations that either lowered PLC-β/IP_3_R abundance or attenuated endolysosomal Ca^2+^ overload restored animal longevity. Collectively, our findings demonstrate that depolarization-dependent regulation of PLC-β–IP_3_R signaling is required for modulation of the ATP/ADP ratio in healthy glutamatergic neurons, whereas hyperactivation of this axis in chronically depolarized glutamatergic neurons shortens animal lifespan by promoting endolysosomal Ca^2+^ overload.

## Significance

We demonstrate that depolarization of *Drosophila* glutamatergic neurons augmented inositol trisphosphate receptor (IP_3_R)-dependent release of endoplasmic reticulum (ER) Ca^2+^, which in turn potentiated mitochondrial Ca^2+^ uptake and ATP production. Perturbations that induced chronic depolarization, including the expression of neurodegeneration-related transgenes, led to the diversion of released ER Ca^2+^ into lysosomes and an attendant shortening of animal lifespan. Thus, genetic disruption of PLC-β–IP_3_R signaling or lysosomal Ca^2+^ uptake restored longevity in animals with chronically depolarized glutamatergic neurons. Our findings point to aberrant Ca^2+^ signaling between the ER and lysosomes as a mechanism by which hyperexcitable glutamatergic neurons shorten animal lifespan.

Spatially circumscribed ATP production at nerve termini is predicated on local mitochondria that are energized when voltage-gated Ca^2+^ channels provide the [Ca^2+^] elevations needed to overcome the low sensitivity of the mitochondrial Ca^2+^ uniporter (MCU) (1–3). In neuronal soma, however, bulk cytosolic [Ca^2+^] is not elevated to levels needed for mitochondrial sequestration. Rather, mitochondrial Ca^2+^ uptake in the somatodendritic compartment occurs at specialized points of contact between mitochondria and endoplasmic reticulum (ER) where Ca^2+^ released by IP_3_Rs is transferred into the mitochondrial matrix (4). Approximately 75 to 90% of the somatic ATP synthesized following interorganellar transfer of Ca^2+^ is consumed by Na^+^/K^+^ ATPases, which help establish resting membrane potential and permit repolarization during activity (5, 6). Therefore, defects in neuronal ATP synthesis result in loss of membrane potential and hyperexcitability (6).

Whether excitability of the somatic plasma membrane (PM) exerts reciprocal influence on mitochondrial [Ca^2+^] and ATP production remains poorly understood. In an attempt to fill some of the gaps in knowledge, we examined the effects of PM potential on mitochondrial ATP production and Ca^2+^ homeostasis in *Drosophila* neurons. Owing to recent reports of neuronal hyperexcitability being a driver of diminished longevity in organisms ranging from *Caenorhabditis elegans* to humans (7–9), we hoped our studies would inform insights into the regulation of aging and lifespan. Moreover, since neuronal hyperexcitability, Ca^2+^ dyshomeostasis, and bioenergetic dysfunction characterize neurodegenerative diseases (6, 10, 11), uncovering actionable molecular targets that bridge these perturbations may bear therapeutic value. Our findings reveal a previously unknown mechanism by which excitability regulates bioenergetics and Ca^2+^ signaling and points to the utility of this signaling circuit in the regulation of longevity.

## Results

### Activation of PLCβ–IP_3_R Signaling in Depolarized *Drosophila* Neurons Increases the [ATP]/[ADP] Ratio in Cell Bodies.

**Fig. 1.**
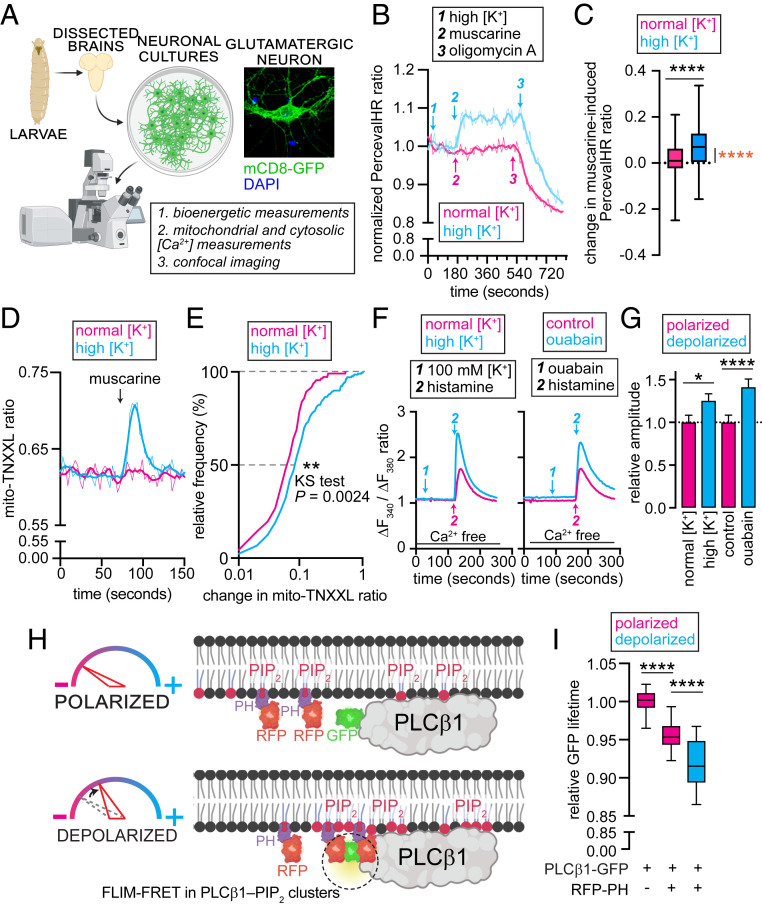
Depolarization augments PLC-β–IP_3_R signaling. (*A*) Experimental workflow used in this study. Neurons were dissociated from third instar larval brains. Live neurons were used for recording bioenergetics and Ca^2+^ signaling, whereas fixed neurons were imaged to examine changes in lysosomes. Image was created with BioRender.com. (*Inset*) Confocal image of a glutamatergic neuron expressing mCD8-GFP. (*B*) Representative traces showing normalized PercevalHR ratio in *Drosophila* glutamatergic neurons depolarized with 51.7 mM [K^+^] (blue) and in normally polarized neurons exposed to a buffer containing 5 mM [K^+^] (pink). Arrows indicate treatments. (*C*) Boxplots quantifying muscarine-induced changes in PercevalHR ratio. Black ****P < 0.0001, Mann–Whitney *U* test; orange ****P < 0.0001, one sample *t* test for a hypothetical mean of 0. (*D*) Representative traces showing mito-TNXXL ratio in depolarized (51.7 mM [K^+^], blue) and polarized (5 mM [K^+^], pink) neurons. Arrow indicates point of muscarine addition. (*E*) Cumulative distribution of muscarine-induced change in mito-TNXXL ratio in depolarized (51.7 mM [K^+^], blue) and polarized (5 mM [K^+^], pink) fly neurons. **P = 0.0024, Kolmogorov–Smirnov test. (*F*) Representative traces showing Fura-2 ratio in N2a cells depolarized with 100 mM [K^+^] (*Left)* or ouabain (*Right*). Arrows indicate treatments. Line indicates Ca^2+^-free bath. (*G*) Bar graphs quantifying normalized amplitudes of Ca^2+^ transients shown in *F*. Values represent mean ± SEM, *P < 0.05, ****P < 0.0001, Mann–Whitney *U* tests. (*H*) PLC-β1–PIP_2_ interactions in depolarized cells are reflected by proximity of PLC-β1-GFP and RFP-PH, which can be detected using FLIM-FRET. (*I*) Boxplots quantifying GFP-lifetime in depolarized or polarized N2a cells expressing the indicated probes. ****P < 0.0001, *t* tests with Bonferroni correction.

We examined the effects of depolarization on cellular bioenergetics and Ca^2+^ homeostasis in *Drosophila* neurons dissociated from third instar larval brains (Fig. 1*A*). In neurons expressing PercevalHR, a reporter of the cytosolic [ATP]/[ADP] ratio (12), we found that the somatic [ATP]/[ADP] ratio was not altered by inhibition of glycolysis using 2-deoxyglucose (2-DG) (neither 2 nor 6 min of 2-DG treatment changed the [ATP]/[ADP] ratio, *SI Appendix*, Fig. S1 *A* and *B*) (13). These findings are consistent with previous reports of glycolysis being more important for ATP production in fly glia than in neurons (14, 15). Despite the insensitivity to 2-DG, the [ATP]/[ADP] ratio diminished rapidly in response to rotenone (inhibitor of complex I of the electron transport chain), oligomycin A (inhibitor of ATP synthase), or FCCP (mitochondrial uncoupler) (*SI Appendix*, Fig. S1 *A* and *B*). Therefore, steady-state [ATP]/[ADP] balance in *Drosophila* neurons depends on mitochondrial oxidative phosphorylation (OXPHOS).

**Fig. 2.**
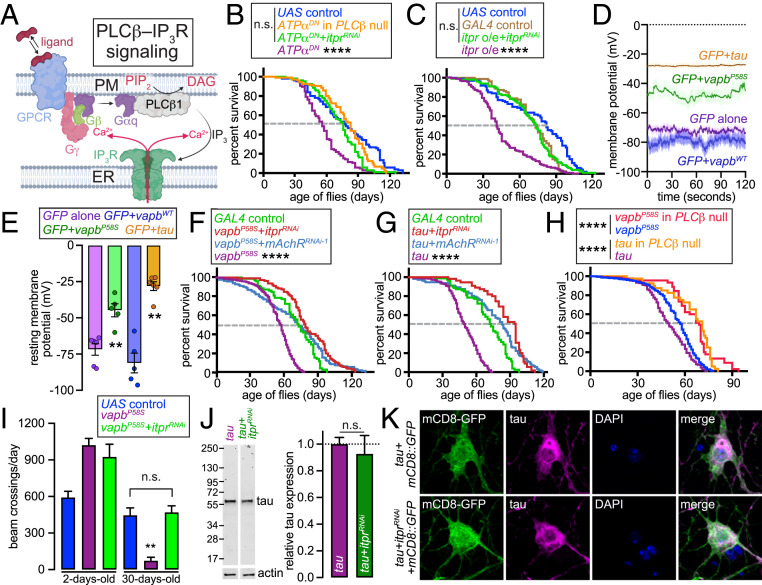
Chronic depolarization of glutamatergic neurons shortens fly lifespan in a PLC-β/IP_3_R-dependent manner. (*A*) Model depicting the PLCβ–IP_3_R signaling cascade. Ligand stimulation of G-αq-coupled receptor (GPCR) causes G-αq to dissociate from G-βγ and activate PLC-β. PLC-β hydrolyzes PIP_2_ to generate DAG and IP_3_. IP_3_ binds to IP_3_R in the ER leading to Ca^2+^ release. Image was created with BioRender.com. (*B* and *C* and *F*–*H*) Lifespan of flies of the indicated genotypes. ****P < 0.0001, log-rank tests with Bonferroni correction. (*D*) Whole-cell recordings in current-clamp mode showing resting membrane potential in dissociated glutamatergic neurons expressing the indicated transgenes. Values represent mean ± SEM. (*E*) Bar graph quantifying the data shown in *D*. Values represent mean ± SEM, **P < 0.005, ANOVA followed by *t* tests with Bonferroni correction. (*I)* Bar graph showing daily locomotion exhibited by adult flies of the indicated genotypes and age. Values represent mean ± SEM of beam-crossing counts. **P < 0.01, ANOVA followed by *t* tests with Bonferroni correction; ns, not significant. (*J*, *Left*) Representative Western blot showing larval brain extracts derived from animals of genotypes indicated on the top probed with antibodies against tau and actin. (*Right*) Bar graph showing quantification of the Western blot. Values represent mean ± SEM; ns, not significant; *t* test. (*K*) Confocal images showing *Drosophila* glutamatergic neurons expressing the transgenes indicated on the left.

**Fig. 3.**
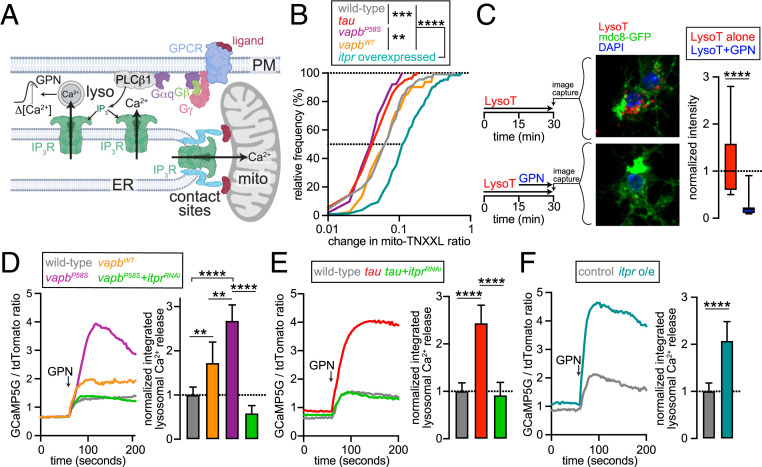
ALS and tauopathy-related transgenes perturb interorganellar transfer of Ca^2+^ and induce endolysosomal Ca^2+^ overload. (*A*) Model showing that Ca^2+^ released via IP_3_Rs can be sequestered into mitochondria and/or endolysosomes. Endolysosomal [Ca^2+^] can be inferred from changes in [Ca^2+^] (Δ[Ca^2+^]) after application of GPN. Image was created with BioRender.com. (*B*) Cumulative distribution of muscarine-induced change in mito-TNXXL ratio in fly neurons of the indicated genotypes. **P < 0.01, ***P < 0.001, ****P < 0.0001, Kolmogorov–Smirnov test. (*C*) Application of GPN led to ablation of LysoTracker (LysoT) staining in fly glutamatergic neurons. Overview of experimental strategy, confocal images of LysoTracker stained neurons, and quantification of LysoTracker intensities are shown. Boxplot quantifies LysoTracker intensity. ****P < 0.0001 Mann–Whitney *U* test. (*D*–*F*, *Left*) Representative traces showing GCaMP5G/tdTomato ratio in dissociated fly glutamatergic neurons expressing the indicated transgenes. Arrow indicates point of GPN application. (*Right*) Bar graphs quantifying GPN-induced changes. Data represent median and 95% CI. **P < 0.005, ****P < 0.0001, Mann–Whitney *U* tests with Bonferroni corrections.

Next, we asked whether the [ATP]/[ADP] ratio was dependent on PM potential and/or ER Ca^2+^ release. While depolarization alone did not elicit changes in the somatic [ATP]/[ADP] ratio (Fig. 1*B*, blue trace 1, and *SI Appendix*, Fig. S1*C*), subsequent application of muscarine—an agonist of PLC-β–coupled metabotropic acetylcholine receptors (mAChRs) (16)—significantly increased the ratio (Fig. 1*B*, blue trace 2, Fig. 1*C*, and *SI Appendix*, Fig. S1*C*). This effect of muscarine was absent in electrically polarized neurons (Fig. 1*B*, pink trace 2, and Fig. 1*C*). Oligomycin A abolished the differences in depolarization- and muscarine-induced changes in the [ATP]/[ADP] ratio (Figs. 1 *B*–*1*, *2*, and *3*, and *SI Appendix*, Fig. S1*D*). Given the requirement for matrix [Ca^2+^] in mitochondrial ATP production (2, 17, 18), we asked whether the increase in the [ATP]/[ADP] ratio following the coincidence of muscarine and high [K^+^] was accompanied by elevation in mitochondrial [Ca^2+^]. Using a mitochondrial Ca^2+^ reporter, mito-TNXXL (18), we found that both the fraction of glutamatergic neurons exhibiting mitochondrial [Ca^2+^] elevations and their response amplitudes were significantly larger upon concurrence of high [K^+^] and muscarine (Fig. 1 *D* and *E*). Taken together, these data suggest that coincident depolarization and activation of PLC-β–coupled receptors was needed for mitochondrial Ca^2+^ uptake and OXPHOS-dependent increase in the [ATP]/[ADP] ratio. Notably, neither stimulus in isolation elicited significant changes in mitochondrial [Ca^2+^] or the [ATP]/[ADP] balance.

### PM Depolarization Potentiates PLCβ–IP_3_R Signaling.

To assess the involvement of cytosolic Ca^2+^ in the interplay between depolarization and mitochondrial [Ca^2+^], we examined changes in cytosolic [Ca^2+^] by expressing the GCaMP5G–tdTomato cytosolic Ca^2+^ sensor (19). As with the [ATP]/[ADP] ratio and mitochondrial [Ca^2+^], coincidence of depolarization and muscarine was needed for significant cytosolic Ca^2+^ signals in terms of the number of responding cells and their response amplitudes (*SI Appendix*, Fig. S1*E*, blue trace 2, and *SI Appendix*, Fig. S1*F*). Neither depolarization nor muscarine alone significantly elevated somatic [Ca^2+^] (*SI Appendix*, Fig. S1*E*, blue trace 1 and pink trace 2, respectively). Amplitudes of Ca^2+^ transients evoked by thapsigargin-mediated ER store depletion, however, were indistinguishable in depolarized or normally polarized cell bodies (*SI Appendix*, Fig. S1*G*), which indicated that the larger Ca^2+^ transients evoked by muscarine in depolarized neurons did not reflect an increase in ER Ca^2+^ content.

#### 

What explains our finding that depolarization augments muscarine-induced Ca^2+^ transients? Stimulation of mAchR results in the release of ER Ca^2+^ via IP_3_Rs (Fig. 2*A*) (20). Since IP_3_Rs reside in the ER membrane (Fig. 2*A*), they cannot be directly gated by PM depolarization. Localization of PLC-β at the PM (20) (Fig. 2*A*), however, raised the possibility that IP_3_ production could be modulated by membrane potential. Using IP_3_R-dependent Ca^2+^ release as a proxy for IP_3_ levels, we examined the effects of depolarization on PLC-β activity. To ensure that evoked Ca^2+^ transients reflected release from ER, we sought to mitigate the cofounding effects of Ca^2+^ entry by excluding Ca^2+^ from the extracellular media. Unfortunately, ER Ca^2+^ transients in *Drosophila* neurons were abolished in the absence of extracellular Ca^2+^. Thus, we turned to Neuro2a (N2a) cells (21), which retained signaling in Ca^2+^-free media, and exhibited significantly larger ER release transients in response to a GPCR–PLC-β agonist when depolarized with either high [K^+^] or the Na^+^/K^+^ ATPase blocker, ouabain (22) (Fig. 1 *F* and *G*). These data indicate that signaling via the PLCβ–IP_3_R pathway is augmented by depolarization.

We previously showed that depolarization increases the propensity for the PLC-β substrate, phosphatidylinositol- (4, 5)-bisphosphate (PIP_2_), to form nanoclusters at the PM (23). PIP_2_ clustering is deduced from examining the proximity between RFP- and GFP-tagged variants of the PIP_2_-sensor, PH-PLC-δ, using fluorescence lifetime imaging-Förster resonance energy transfer (FLIM-FRET) (23, 24) (*SI Appendix*, Fig. S2*A*). Shorter GFP lifetimes in cells expressing RFP-PH and GFP-PH indicate enhanced clustering. Consistent with the effects of high [K^+^] (23), ouabain significantly decreased GFP lifetime in N2a cells coexpressing RFP- and GFP-PH (*SI Appendix*, Fig. S2 *B* and *C*). Next, we asked whether depolarization influences the extent of association between PIP_2_ and PLC-β. To this end, we examined FLIM-FRET between PLC-β1-GFP and RFP-PH (Fig. 1*H*). Cells coexpressing PLC-β1-GFP and RFP-PH had lower GFP lifetimes than did cells expressing PLC-β1-GFP alone, suggesting constitutive PIP_2–_PLC-β1 association (Fig. 1*I*). Depolarization further reduced GFP lifetime in PLCβ1-GFP and RFP-PH coexpressing cells (Fig. 1*I*). Thus, depolarization augments PIP_2–_PLC-β1 association such that subsequent stimulation of PLC-β activity would result in elevated IP_3_ production and IP_3_R-mediated ER Ca^2+^ release.

### Chronic Depolarization of Glutamatergic Neurons Shortens the Lifespan of Adult *Drosophila* via Hyperactivation of PLC-β–IP_3_R Signaling.

Hyperexcitability of glutamatergic neurons induces premature aging and shortens lifespan in many organisms (7, 8). Since inhibition of the Na^+^/K^+^ ATPase potentiated PLC-β–IP_3_R signaling in N2a cells (Fig. 1 *F* and *G*), we reasoned that genetic inhibition of pump activity in flies could uncover the involvement of PLC-β–IP_3_R signaling in the relationship between excitability and longevity. To test this model, we first used a dominant-negative α-subunit of Na^+^/K^+^ ATPase (ATP-α^DN^) (25), which abbreviated fly lifespan when expressed in glutamatergic neurons (Fig. 2*B*). Either the concomitant knockdown of *IP*_*3*_*R* [*itpr*, decreased *itpr* messenger RNA (mRNA) upon *itpr*^*RNAi*^ expression is shown in *SI Appendix*, Fig. S3*A*] or deletion of *PLC*-*β* (*norpA*) restored the lifespan of flies expressing *ATP*-*α*^*DN*^ (Fig. 2*B* and *SI Appendix*, Fig. S3*B*). The additional finding that *IP*_*3*_*R* knockdown in isolation did not extend lifespan in controls (*SI Appendix*, Fig. S3*C*) implicated the PLC-β–IP_3_R pathway in the early lethality apparent in *ATP*-*α*^*DN*^*–*expressing animals. In agreement, overexpression of *IP*_*3*_*R* in glutamatergic neurons was sufficient to shorten animal lifespan (Fig. 2*C*).

Given the onset of cell-intrinsic neuronal hyperexcitability in neurodegenerative diseases (10, 11), we probed the relationship between PM potential, PLC-β–IP_3_R signaling, and lifespan in fly models of tauopathy and amyotrophic lateral sclerosis (ALS). Using whole-cell patch-clamp recordings, we first confirmed that glutamatergic neurons expressing an ALS-causing variant of *vapb* (*vapb*^*P58S*^) or human *tau* (26–29) were constitutively depolarized relative to neurons expressing either *GFP* alone or wild-type *vapb* (*vapb*^*WT*^) (Fig. 2 *D* and *E*). Concordantly, glutamatergic expression of *vapb*^*P58S*^ or *tau* shortened animal lifespan (Fig. 2 *F* and *G* and *SI Appendix*, Fig. S3*D*). Although the expression of *vapb*^*WT*^ also shortened animal lifespan, effects of this transgene were mild compared to those evoked by *vapb*^*P58S*^ (*SI Appendix*, Fig. S3*D*). Concomitant knockdown of either *IP*_*3*_*R* or *mAchR* (*SI Appendix*, Fig. S3*A*) or the deletion of *PLC*-*β* prevented the premature demise induced by *vapb*^*P58S*^ or *tau* (Fig. 2 *F*–*H* and *SI Appendix*, Fig. S3 *E* and *F*). Age-dependent decline in ambulation in animals expressing *vapb*^*P58S*^ was also ameliorated by *IP*_*3*_*R* knockdown (Fig. 2*I*).

To validate the protective effects of *IP*_*3*_*R* knockdown, we first determined whether coexpression of *UAS*-*itpr*^*RNAi*^ could dilute GAL4-induced gene expression. Abundance of neutral protein (mCD8-GFP, encoded by *UAS*-*mCD8*-*GFP*) was not altered by the coexpression of *UAS*-*itpr*^*RNAi*^ (*SI Appendix*, Fig. S3*G*). Similarly, coexpression of *itpr*^*RNAi*^ changed neither the abundance nor the distribution of ectopic human tau in glutamatergic neurons (Fig. 2 *J* and *K*). These data rule out GAL4 dilution or alterations in transgene expression. Arguing against off-target effects, we found that overexpression of *IP*_*3*_*R* complimentary DNA (cDNA) prevented *itpr*^*RNAi*^ from restoring the lifespan of *vapb*^*P58S*^- or *tau*-expressing animals (*SI Appendix*, Fig. S3 *H* and *I*). Lethality induced by concomitant expression of both *vapb*^*P58S*^ and *tau* was also mitigated by *IP*_*3*_*R* knockdown (*SI Appendix*, Fig. S3*I*), which indicates that both transgenes exert their effects via IP_3_R. Together, these data point to a critical role for the mAchR–PLC-β–IP_3_R pathway (Fig. 2*A*) in the precocious lethality stemming from transgenes that induce chronic neuronal depolarization.

#### 

Do our findings suggest a general role for ER Ca^2+^ release in the regulation of fly lifespan? Reducing ryanodine receptor (*RyR*) gene dosage, which robustly reduces RyR-mediated ER Ca^2+^ release (19, 30), did not extend lifespan of animals expressing *vapb*^*P58S*^ or *tau* (*SI Appendix*, Fig. S4 *A* and *B*). Furthermore, animals expressing both *tau* and an RNA interference (RNAi) line against *RyR* (*RyR*^*RNAi*^) exhibited significantly shorter lifespan than did the animals expressing *RyR*^*RNAi*^ alone (*SI Appendix*, Fig. S4*C*). This result was in contrast to the finding that animals expressing both *tau* and *itpr*^*RNAi*^ lived even longer than did the flies expressing *itpr*^*RNAi*^ alone (*SI Appendix*, Fig. S4*C*), and thus, demonstrated the inability of *RyR* knockdown to suppress tau-induced lethality. Finally, increased *RyR* dosage, which promotes the release of Ca^2+^ through these channels (31, 19), did not shorten fly lifespan (*SI Appendix*, Fig. S4*D*). Taken together, these data point to a role for IP_3_R, but not RyR, in the regulation of fly longevity.

### Endolysosomal Ca^2+^ Overload Occurs in Response to PLC-β–IP_3_R Hyperactivity in *Drosophila* Glutamatergic Neurons.

IP_3_Rs have well-established roles in interorganellar Ca^2+^ transfer between the ER and organelles such as the mitochondria and endolysosomes (Fig. 3*A*) (32–36). Indeed, we found that overexpression of *IP*_*3*_*R* was sufficient to augment mitochondrial [Ca^2+^] elevation in response to mAchR activation by muscarine (Fig. 3*B*). Since *vapb*^*P58S*^ or *tau* led to chronic depolarization and increased PLC-β–IP_3_R activity, we speculated that these transgenes would also promote mitochondrial Ca^2+^ uptake. However, muscarine-induced mitochondrial Ca^2+^ uptake was diminished in *vapb*^*P58S*^- or *tau*-expressing neurons compared to those that were wild type or expressed *vapb*^*WT*^ (Fig. 3*B*). Therefore, while overexpression of *IP*_*3*_*R* led to mitochondrial Ca^2+^ overload, IP_3_R-dependent mitochondrial Ca^2+^ uptake was compromised upon expression of either *vapb*^*P58S*^ or *tau*.

To determine the consequences of altered mitochondrial Ca^2+^ uptake on lifespan, we knocked down the MCU, which participates in Ca^2+^ transfer into the matrix (*SI Appendix*, Fig. S3*A*) (37, 38). Confirming the notion that mitochondrial Ca^2+^ overload can be toxic, concomitant *MCU* knockdown significantly extended the lifespan of flies overexpressing *IP*_*3*_*R* in glutamatergic neurons (*SI Appendix*, Fig. S5*A*). In contrast, *MCU* knockdown further worsened the survival of flies expressing *vapb*^*P58S*^ (*SI Appendix*, Fig. S5*B*), while lifespan of *tau*-expressing animals was not altered by this manipulation (*SI Appendix*, Fig. S5*C*). These data indicate that although *IP*_*3*_*R* overexpression shortens lifespan via mitochondrial Ca^2+^ overload, the PLC-β–IP_3_R pathway contributes to the lifespan of *vapb*^*P58S*^- or *tau*-expressing animals via a distinct mechanism.

#### 

Could IP_3_R-dependent transfer of Ca^2+^ into endolysosomes explain the effects of *vapb*^*P58S*^ or tau? Endolysosomal [Ca^2+^] can be inferred from cytosolic transients triggered by the lysoosmolytic agent glycyl-l-phenylalanine-2-naphthylamide (GPN) (Fig. 3*A*) (39, 40). Consistent with reports of GPN ablating LysoTracker-positive endolysosomes (41), we found that GPN dramatically lowered LysoTracker staining in dissociated glutamatergic neurons (Fig. 3*C*). Arguing against the possibility that GPN directly releases ER Ca^2+^ (42), thapsigargin-sensitive ER Ca^2+^ stores were not diminished by GPN pretreatment (*SI Appendix*, Fig. S5*D*). Inhibition of the vacuolar ATPase using Bafilomycin A1 (BafA1) also depletes endolysosomal Ca^2+^, albeit via an unknown leak mechanism (43, 44). Upon BafA1 pretreatment, GPN-induced transients were significantly stunted (*SI Appendix*, Fig. S5*E*), which indicates overlap between the Ca^2+^ stores targeted by BafA1 and GPN. Together, our data support the prevailing view that GPN induces lysosomal rupture and the acute release of Ca^2+^ stored in those vesicles.

GPN-induced Ca^2+^ transients were significantly larger in neurons expressing *vapb*^*P58S*^ or *tau* (Fig. 3 *D* and *E*). Although endolysosomal [Ca^2+^] was partially elevated in neurons overexpressing *vapb*^*WT*^, vesicular [Ca^2+^] was even higher in *vapb*^*P58S*^-expressing neurons (Fig. 3*D*). Concomitant knockdown of *IP*_*3*_*R* prevented the increase in GPN-induced endolysosomal Ca^2+^ release in *vapb*^*P58S*^- or *tau*-expressing neurons (Fig. 3 *D* and *E*). Expression of *ATP*-*α*^*DN*^ also augmented GPN-sensitive endolysosomal Ca^2+^ stores in an IP_3_R-dependent manner (*SI Appendix*, Fig. S5*F*). Conversely, GPN-induced Ca^2+^ transients were augmented by *IP*_*3*_*R* overexpression (Fig. 3*F*). These changes in endolysosomal [Ca^2+^] did not reflect alterations in vesicle biogenesis because LysoTracker staining revealed no correlation between the response to GPN and the number of endolysosomes per cell (*SI Appendix*, Fig. S5*G*). These data are consistent with a requirement for IP_3_Rs in endolysosomal Ca^2+^ sequestration.

### Endolysosomal Ca^2+^ Overload Requires TRPML and Shortens Fly Lifespan.

**Fig. 4.**
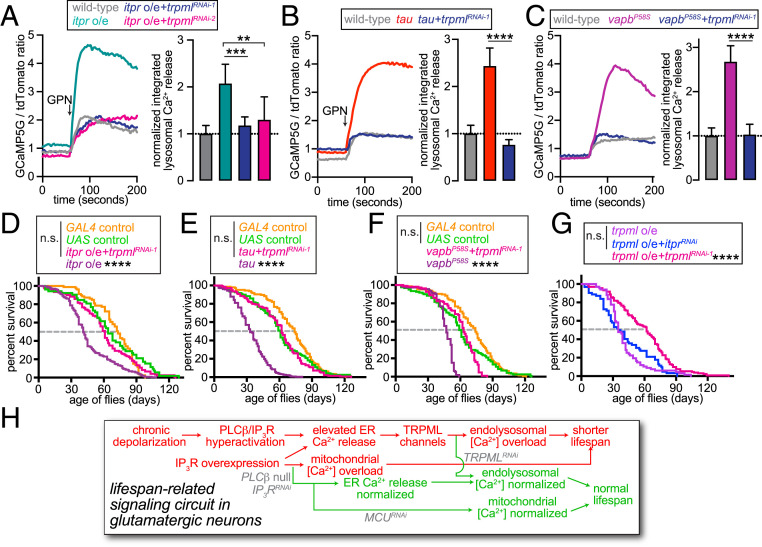
TRPML operates downstream of IP_3_Rs in the regulation of endolysosomal [Ca^2+^] and lifespan. (*A*–*C*, *Left*) Representative traces showing GCaMP5G/tdTomato ratio in dissociated fly glutamatergic neurons expressing the indicated transgenes. Arrow indicates point of GPN application. (*Right*) Bar graphs quantifying GPN-induced changes in GCaMP5G/tdTomato ratio in dissociated fly glutamatergic neurons expressing the indicated transgenes. Data represent median and 95% CI. **P < 0.005, ***P < 0.001, ****P < 0.0001, Mann–Whitney *U* tests with Bonferroni corrections. (*D–G*) Lifespan of flies of the indicated genotypes. ****P < 0.0001, log-rank tests with Bonferroni correction. (*H*) Model depicting the signaling circuit that relates depolarization of glutamatergic neurons with endolysosomal [Ca^2+^] overload and regulation of lifespan.

We reasoned that concomitant knockdown of the endolysosomal cation channel, TRPML (43, 45, 46), would further elevate vesicular [Ca^2+^] in neurons overexpressing *IP*_*3*_*R*. However, knockdown of *trpml* using either one of two independent RNAi lines (*SI Appendix*, Fig. S3*A*) mitigated IP_3_R-induced endolysosomal Ca^2+^ overload (Fig. 4*A*). Knockdown of *trpml* also reduced the size of GPN-induced Ca^2+^ transients in neurons expressing either *tau* or *vapb*^*P58S*^ (Fig. 4 *B* and *C*) but not in control neurons (*SI Appendix*, Fig. S6*A*). Conversely, overexpression of *trpml* augmented vesicular [Ca^2+^] (*SI Appendix*, Fig. S6*A*). As was the case with knockdown of *IP*_*3*_*R*, knockdown of *trpml* did not significantly alter the abundance of ectopic tau (*SI Appendix*, Fig. S6*B*). Thus, diminished perturbagen abundance did not underlie the observed restoration of vesicular [Ca^2+^]. Since IP_3_R-dependent augmentation of the GPN responses was not influenced by coexpression of a neutral transgene (*luciferase*) (*SI Appendix*, Fig. S6*C*), we also ruled out the involvement of GAL4 dilution.

Pointing to the correlation between endolysosomal [Ca^2+^] and lifespan, knockdown of *trpml* restored longevity in animals that overexpressed *IP*_*3*_*R*, *tau*, or *vapb*^*P58S*^ in glutamatergic neurons (Fig. 4 *D*–*F*). Conversely, overexpression of *trpml* in glutamatergic neurons shortened animal lifespan (*SI Appendix*, Figs. S6*D* and S4*G*). Although the effect of *trpml* overexpression on lifespan was countered by *trpml* knockdown, concomitant knockdown of *IP*_*3*_*R* did not confer similar benefits in those animals (*SI Appendix*, Figs. S6*D* and S4*G*). Thus, *trpml* is epistatic to *IP*_*3*_*R* in the sense that increased TRPML abundance bypassed the beneficial effects of *IP*_*3*_*R* knockdown. In summary, TRPML is an intermediary in the effects of IP_3_R on endolysosomal [Ca^2+^] and lifespan.

## Discussion

Our data reveal a signaling circuit that actuates the relationship between glutamatergic excitability and organismal lifespan (Fig. 4*H*) (7, 8). The crux of this model is our finding that depolarization increases the gain of PLC-β–IP_3_R signaling. We envision that the ostensible purpose of elevated IP_3_R activity in depolarized neurons is enhanced mitochondrial Ca^2+^ uptake and ATP production. Newly synthesized ATP could then be used to restore cellular electrochemical balance. Dominant-negative ATP-α, however, rendered membrane potential unresponsive to ATP production and resulted in shorter lifespan via a process requiring PLC-β–IP_3_R signaling (red text, Fig. 4*H*). Consequently, genetic attenuation of PLC-β–IP_3_R activity restored animal lifespan (green text, Fig. 4*H*). Expression of *vapb*^*P58S*^ or *tau* also led to chronic depolarization of neurons and PLC-β–IP_3_R-dependent shortening of lifespan, which agrees with prior reports of increased IP_3_R abundance or activity in models of neurodegeneration (47–49). Interestingly, Ca^2+^ transfer between ER and mitochondria was disrupted in neurons expressing *vapb*^*P58S*^ or *tau*, which in principle, would be expected to limit OXPHOS-dependent ATP production and Na^+^/K^+^ ATPase activity as described (6). However, whole-cell recordings of neurons expressing these transgenes suggest additional complexity. Because cytosolic [K^+^] in whole-cell recordings was clamped by the pipette solution, a role for Na^+^/K^+^ ATPases in setting the membrane potential was obviated. Therefore, chronic depolarization observed in *vapb*^*P58S*^- or *tau*-expressing neurons likely reflected perturbations additional to diminished Na^+^/K^+^ ATPase activity; for instance, decreased activity or abundance of Na^+^ or K^+^ channels (50, 51). Nevertheless, the relationship between membrane potential and PLC-β–IP_3_R signaling was agnostic to the trigger that led to sustained depolarization, which implies that our findings would be applicable to other conditions associated with chronic neuronal depolarization.

Although *IP*_*3*_*R* overexpression shortened lifespan via attendant mitochondrial Ca^2+^ overload, it was endolysosomal Ca^2+^ overload that led to early demise of animals expressing *vapb*^*P58S*^ or *tau* (Fig. 4*H*). Knockdown of *trpml*, which encodes an endolysosomal Ca^2+^ and Na^+^ channel (43, 45, 46), restored both vesicular [Ca^2+^] and lifespan downstream of IP_3_Rs (Fig. 4*H*). Despite reports of TRPML1 activity being elevated in models of neurodegeneration (52), roles for a cation release channel in Ca^2+^ uptake was surprising. One explanation for these data are that TRPML-dependent endolysosomal biogenesis via the purported Ca^2+^–calcineurin–TFEB axis (53) could increase the number of GPN-sensitive vesicles, and therefore, result in elevated Ca^2+^ mobilization. However, none of the genetic manipulations that influenced vesicular [Ca^2+^] concomitantly altered the number of endolysosomal vesicles in those cells. Therefore, we favor the alternative model that TRPML is required for positioning endolysosomes in perinuclear regions abutting the ER in order to permit uptake of Ca^2+^ released by IP_3_Rs (36, 54). Indeed, mammalian TRPML1 is needed to stabilize interactions between ER and lysosomes prior to Ca^2+^ transfer between the two organelles (36). Although additional studies would be needed for understanding the roles of TRPML in endolysosomal Ca^2+^ uptake and the regulation of longevity, our findings indicate that function of this endolysosomal channel in glutamatergic neurons is a determinant of organismal lifespan.

## Materials and Methods

### Fly Husbandry.

Flies were reared at 21 °C on standard fly food (1 L of food contained 95 g agar, 275 g brewer’s yeast, 520 g of cornmeal, 110 g of sugar, 45 g of propionic acid, and 36 g of tegosept). The following fly lines were obtained from Bloomington *Drosophila* Stock Center: *vglut*^*ok371*^-*GAL4* (*ok371*-*GAL4*) (55), *d42*-*GAL4* (56), *hs*-*GAL4* (RRID:BDSC_2077), *RyR*^*16*^ (30), *UAS*-*tau* (*UAS*-*MAPT.A*; *htau*^*ON3R*^) (29), *UAS*-*itpr* (*UAS*-*Itp*-*r83A.V*) (19, 57), *UAS*-*itpr*^*RNAi*^ (*TRiP*.*JF01957*) (19), *UAS*-*trpml*^*RNAi-1*^ (*TRiP*.*JF01466*), *UAS*-*trpml*^*RNAi-2*^ (*TRiP*.*JF01239*), *UAS*-*mAChR*-*A*^*RNAi-1*^ (*TRiP*.*HMC02343*), *UAS*-*mAChR*-*A*^*RNAi-2*^ (*TRiP*.*JF02725*), *UAS*-*MCU*^*RNAi*^ (*TRiP*.*HMS05618*), *UAS*-*RyR*^*RNAi*^ (*TRiP*.*HM05130*), *UAS*-*luc* (*UAS*-*luc*-*VALIUM10*), and *UAS*-*mCD8*-*GFP*. Other strains used in the study were as follows: *UAS*-*trpml* (46), *UAS*-*PercevalHR* (described below), *UAS*-*mito*-*TNXXL* (*UAS*-*2mt8TNXXL*; gift from Dr. Gregory Macleod, Florida Atlantic University, Boca Raton, FL) (18), *UAS*-*tdTomato*-*2A*-*GCaMP5G* (19), *UAS*-*vapb*^*WT*^ and *UAS*-*vapb*^*P58S*^ (gifts from Dr. Hugo J. Bellen, Baylor College of Medicine, Houston, TX) (28), *canton S*, *Oregon R*, *w*^*1118*^, *norpA*^*P33*^ and *norpA*^*P54*^ (58), *UAS*-*ATP*-*α*^*D369N*^ (gift from Dr. Leslie C. Griffith) (25), and *RyR*^*24D03*^ (31, 19).

### Construction of *UAS*-*PercevalHR.*

PercevalHR was PCR amplified from Addgene construct No. 49082 (https://www.addgene.org/49082/) and cloned into *pUAST*-*C5* via an intermediate pC5-Kan shuttle vector using *Not I* and *Xho I* restriction sites. The insert was fully sequenced before submission to Bestgene for random P-element–based integration.

### Mammalian Cell Culture and Dissociation of *Drosophila* Neurons.

N2a cells were cultured in Dulbecco's modified Eagle medium (DMEM; D5796; Sigma-Aldrich) supplemented with 10% fetal bovine serum (FBS) and maintained at 5% CO_2_ and 37 °C. For dissociation and culture of primary glutamatergic neurons from *Drosophila*, we adopted previously described protocols for isolating and culturing fly neurons (59–61). Briefly, the exterior of wandering third instar larvae was sterilized by brief submersion in ethanol and then washed with sterilized H_2_O before dissection in filtered Schneider’s medium (S0146; Sigma-Aldrich) containing 10% FBS, antibiotic/antimycotic solution (A5955; Sigma-Aldrich), and 50 μg/mL of insulin (I6634; Sigma-Aldrich). Brains dissected from these larvae were washed in separate wells containing filtered Schneider’s medium before being transferred to a filtered HL-3 solution (70 mM NaCl, 5 mM KCl, 1 mM CaCl_2_, 20 mM MgCl_2_, 10 mM NaHCO_3_, 115 mM sucrose, 5 mM trehalose, and 5 mM Hepes) supplemented with 0.423 mM l-cysteine (Calbiochem) and 5 U/mL papain (Worthington) (Note: after l-cysteine addition but before papain addition, the pH of the solution was recalibrated to 7.4). The brains were then enzymatically digested in the papain solution for 20 min before transfer to a 1.5 mL tube containing 1 mL of filtered Schneider’s medium. Cells were centrifuged at 100 G for 1 min prior to decantation of Schneider’s medium. The solution was replaced with 1 mL of fresh filtered Schneider’s medium. This process was repeated twice before neurons were dissociated by pipetting repeatedly until the solution was homogeneous. The solution with dissociated neurons was then placed on 35 mm glass-bottom dishes (D35-10-0-N; Cellvis) that had been coated with concanavalin A (C2010; Sigma-Aldrich). Cells were cultured in Schneider’s medium supplemented with 10% FBS, antibiotic/antimycotic solution (A5955; Sigma-Aldrich), and 50 μg/mL of insulin (I6634; Sigma-Aldrich) at room temperature in a humidified container at room temperature. After each day in culture, cells were washed twice with phosphate buffered saline (PBS) to remove any yeast contamination or debris remaining from dissociation. Dissociated neurons were used for experiments 4 d after preparation but remained healthy in cultures for >10 d.

### Live-Cell Imaging in Fly Primary Neurons.

#### Mito-TNXXL.

Culture media was replaced with HL-3 (70 mM NaCl, 5 mM KCl, 1 mM CaCl_2_, 20 mM MgCl_2_, 10 mM NaHCO_3_, 115 mM sucrose, 5 mM trehalose, and 5 mM Hepes; pH 7.2, room temperature). Mito-TNXXL signals, which represent free mitochondrial matrix [Ca^2+^], were recorded by measuring CFP and cpCitrine emissions (18). Briefly, emissions at 482 nm and 532.5 nm were monitored after excitation at 445 nm using an A1R laser confocal microscope with a 40× objective (Nikon). Background emission signals, which were subtracted from all captured images, were measured from a cell-free region of interest (ROI). For experiments requiring neurons with fully polarized membrane potential, the baseline mitochondrial [Ca^2+^] was established by recording cells for 1 min before addition of muscarine (1 mM). To depolarize the neurons, the bath was replaced with high [K^+^] HL-3 (23.3 mM NaCl, 51.7 mM KCl, 1 mM CaCl_2_, 20 mM MgCl_2_, 10 mM NaHCO_3_, 115 mM sucrose, 5 mM trehalose, and 5 mM Hepes; pH 7.2), which as per the Goldman–Hodgkin–Katz equation, would depolarize the membrane potential of fly neurons to ∼ −20 mV from the empirically determined values of −75 mV (Fig. 2 *D* and *E*). Muscarine (1 mM) was then added 2 min after depolarization and mito-TNXXL signals were recorded for 3 min. In the traces plotting the mito-TNXXL ratios against time (Fig. 1*D*), we show both the raw data (thin lines) and smoothed values (thick lines). We used the smoothing function available in Prism 8, which averaged four neighbors on either side of each value, and a second order smoothing polynomial. The amplitude of the cpCitrine/CFP ratio represented free [Ca^2+^] in mitochondrial matrix (18). For quantification of responses, magnitudes of responses from baseline were calculated by dividing the maximum value measured following muscarine stimulation by the average of the five baseline values immediately preceding muscarine addition. A total of >50 cells from a minimum of four independently conducted experiments was completed per genotype/condition.

#### PercevalHR.

Culture media was replaced with HL-3. PercevalHR signals were recorded by measuring the ratio of fluorescence emissions at 525 nm sequentially excited at 487.5 nm and 407.8 nm. An A1R laser confocal microscope with 40× objective (Nikon) was used for measurement. Background emission signals were measured from a cell-free ROI. Baselines were established for 1 min before addition of muscarine (1 mM). For cells subjected to depolarization, the bath was replaced with high [K^+^] HL-3. Muscarine (1 mM) was then added 2 min after depolarization, and PercevalHR signals were recorded for 3 min. We added 2-DG (10 mM), rotenone (100 μM), oligomycin A (10 μM), and FCCP (100 μM) as needed, and signals were recorded. Amplitudes of the emission ratio represented the cytosolic [ATP]/[ADP] ratio (12). Data were quantified as change in muscarine-induced PercevalHR ratio. In the traces plotting the PercevalHR ratios against time (Fig. 1*B* and *SI Appendix*, Fig. S1 *A* and *C*), we show both the raw data (thin lines) and smoothed values (thick lines). We used the smoothing function available in Prism 8, which averaged four neighbors on either side of each value, and a second order smoothing polynomial. For quantification purposes, we used custom R code to calculate the maximum change or the integrated change [i.e., area under the curve for two minutes after addition of the relevant drug using the area under the curve (AUC) function in R] in the ratio following the application of muscarine. A total of >80 cells from a minimum of five independently conducted experiments for each condition were used for the calculations.

#### GCaMP5G*-*tdTomato.

Culture media was replaced with HL-3. GCaMP5G and tdTomato were sequentially excited at 488 nm and 561 nm, respectively, by an A1 laser confocal microscope with a 40× objective (Nikon). Emission signals at 525 nm and 595 nm were recorded. Backgrounds were measured from a cell-free ROI. Baselines were established for 1 min before addition of muscarine (1 mM). For cells subjected to depolarization, the bath was replaced with high [K^+^] HL-3. Muscarine (1 mM) was then added 2 min after depolarization and GCaMP5G-tdTomato signals were recorded for 3 min. Total ER [Ca^2+^] was quantified as the change in cytosolic Ca^2+^ signals following store depletion with the SERCA inhibitor, thapsigargin, as described (19). Amplitudes of the GCaMP5G/tdTomato ratio represents cytosolic free [Ca^2+^] (19). Amplitudes were quantified as change in ratio from baseline and calculated using custom code generated using R. A total of >50 cells from a minimum of five independently conducted experiments for each condition were used for the calculations.

#### Lysosomal Ca^2+^ release.

Supplemented Schneider’s medium used for maintaining cells was replaced with HL-3. Baseline GCaMP5G/tdTomato ratios were recorded for 1 min before the bath was replaced with Ca^2+^-free HL-3. The cells were then recorded for an additional 1 min before treatment with GPN (500 μM). In the case of cells pretreated with BafA1, 400 nM BafA1 was added to the cells, and GPN was then applied ∼20 min later. GPN-induced responses were quantified as integrated change in the ratio (i.e., area under the curve for a duration of 2 min following GPN application calculated using the AUC function in R). A total of >45 cells from a minimum of 5 independently conducted experiments for each condition were used for the calculations.

### Cytosolic Ca^2+^ Imaging in N2a Cells.

Fura-2 signals, which represent cytosolic free [Ca^2+^], were recorded by detecting intensities of emission at 510 nm after excitation at 340 and 380 nm using a Nikon TiE wide-field fluorescence imaging system (Nikon). The background subtracted emission ratio (ΔF_340_/ΔF_380_) was measured and calculated by NIS Elements imaging software (Nikon). Cells were loaded with 10 μM fura-2 (Invitrogen) for 30 min in culture medium at 37 °C. Cells were then washed and bathed in a bath solution containing 140 mM NaCl, 5 mM KCl, 2 mM CaCl_2_, 1 mM MgCl_2_, 10 mM Hepes, 10 mM glucose, and 30 mM sucrose; pH 7.4. Baseline fura-2 signals were recorded for 1 min, before replacing the bath with Ca^2+^-free bath solution. To depolarize PM with extracellular K^+^, we changed the concentrations of K^+^ and Na^+^ in the bath solution from 5 mM KCl/140 mM NaCl to 100 mM KCl/45 mM NaCl (Ca^2+^-free). To depolarize PM using ouabain, 50 μM ouabain or 0.2 μL dimethylsulfoxide (DMSO, vehicle control) was added into the bath 30 s after Ca^2+^-free bath exchange. Histamine was added 1.5 min after depolarization. The fura-2 signals were recorded for 3 min after histamine application. Amplitude of the ΔF_340_/ΔF_380_ signal represented IP_3_R-dependent ER Ca^2+^ release. A total of >80 cells from seven independently conducted experiments for each condition were used for the calculations.

### Whole-Cell Patch-Clamp Recordings.

Approximately 3 h after plating, GFP-expressing dissociated primary *Drosophila* neurons were identified and selected for whole-cell patch clamp. Before measurement of resting membrane potential, the bath solution was replaced with room temperature HL-3 (70 mM NaCl, 5 mM KCl, 1 mM CaCl_2_, 20 mM MgCl_2_, 10 mM NaHCO_3_, 115 mM sucrose, 5 mM trehalose, and 5 mM Hepes; pH 7.2). The pipette solution used contained the following: 109 mM K-gluconate, 10 mM NaCl, 1.7 mM MgCl_2_, 0.085 mM CaCl_2_, 0.94 mM EGTA, 2 mM ATP, and 8.5 mM Hepes; pH 7.2. Recording pipettes were pulled from micropipette glass (Sutter Instruments) to 8 to 10 M-Ω on a PC-10 puller (Narishige). Clamping was performed with an EPC10 (HEKA Instruments) amplifier. Commands were made from the PatchMaster program (version 2 × 90.1; HEKA). G-Ω seal was achieved under voltage-clamping mode with V membrane holding at −70 mV to prevent cell excitation after the membrane was broken. Cells with resistance higher than 1 G-Ω were used for measuring resting membrane potential. Currents were clamped at zero, and voltage was continuously recorded at 10 kHz for a minimum of 3 min per cell.

### Analysis of Fly Lifespan.

Newly eclosed adult flies were collected and transferred to vials containing standard fly food (≤15 flies per vial). Flies were kept at room temperature (∼21 °C) and transferred to new vials twice a week. Dead flies at the bottom of the old vials were counted after each transfer until all the animals in a cohort died.

### *Drosophila* Activity Assays.

Adult flies were reared at 25 °C in an incubator with a 12 h light-dark cycle. Locomotor activity was recorded using a 32-sample *Drosophila* activity monitor (DAM2; TriKinetics). Individual flies were enclosed in a hollow 65 mm glass rod plugged with food (5% sucrose and 2.5% agar) on one end and cotton on the other. The vials are bisected by a central infrared beam, which reports the number of times each fly crosses the infrared beam per minute. Flies were placed in the monitor at least 2 h before data acquisition. The recordings lasted for 1.5 d, after which the flies were removed from the vials and maintained on standard fly food until the next recording.

### FLIM-FRET Experiments.

N2a cells coexpressing PLC-β1-GFP or GFP-tagged PH-PLC-δ with either empty vector pC1 or RFP-tagged PH-PLC-δ were washed with PBS, fixed in 4% paraformaldehyde, and quenched with 50 mM NH_4_OH. FRET pairs were transfected using standardized protocols to optimize for equal expression of respective proteins. Cells were imaged using a 60× Plan-Apo/1.4NA oil emersion lens mounted on a wide-field Nikon Eclipse microscope. GFP was sinusoidally excited by a modulating 3 watt 497 nm light-emitting diode at 40 MHz, and fluorescence lifetime was measured using a Lambert Instrument (Roden) FLIM unit mounted on the Nikon Eclipse microscope. A total of >60 cells were imaged and lifetime (phase) values were pooled and averaged.

### Western Blotting.

Adult heads or dissected larval brains were harvested and homogenized in 2× Laemmli sample buffer (Bio-Rad). Extracts were then loaded onto 4 to 20% Tris-glycine gels (Bio-Rad) for sodium dodecyl sulfate polyacrylamide gel electrophoresis (SDS-PAGE). After transfer to nitrocellulose membranes, blots were blocked in Odyssey blocking buffer (LI-COR Biosciences). Blots were then incubated with primary and secondary antibodies diluted in Odyssey blocking buffer. Blots were incubated with primary antibodies overnight at 4 °C and with secondary antibodies for 2 h at room temperature. Blots were imaged using the Odyssey imaging platform (LI-COR Biosciences). Quantification of band intensities was done using ImageJ (NIH). Primary antibodies used were mouse monoclonal anti-tau (1:1,000; clone T46, 12-6400, Invitrogen), rabbit anti-actin (1:2,000; Sigma, A2066), mouse anti–α-tubulin (1:1,000; 12G10, DSHB), and rabbit anti-GFP (1:1,000; A-11122, Invitrogen). Secondary antibodies used were goat IRDye 680LT anti-rabbit and goat IRDye 800CW anti-mouse (LI-COR Biosciences) at 1:20,000 and 1:15,000, respectively.

### Confocal Imaging of *Drosophila* Neurons.

#### Immunostaining.

Cultured neurons were fixed in 4% PFA in PBS for 15 min at room temperature. The cells were then washed with PBS containing 0.1% triton-X100 and incubated with primary antibodies (1:250 monoclonal anti-tau) for 1 h before wash and incubation with secondary antibodies (1:10,000 anti-mouse Alexa 568) for another hour. Cells were then washed one more time before being mounted in Vectashield containing DAPI (Vector laboratories). Cells were imaged on Nikon A1 confocal microscopy using a 60× oil objective.

#### LysoTracker staining.

Full growth media over the cultured neurons was replaced with media containing LysoTracker Red DND-99 (1:1,000, L7528, Invitrogen) for 30 min at room temperature. The cells were then washed 3× with PBS and fixed in 4% PFA in PBS for 15 min at room temperature. The cells were then washed with PBS containing 0.1% triton-X100 and mounted in Vectashield containing DAPI (Vector laboratories). To examine GPN-induced lysosomal rupture, we applied GPN to the media containing LysoTracker for the final 15 min before wash and fixation (see schematic in Fig. 3*C*). Cells were imaged on Nikon A1 confocal microscopy using a 60× oil objective. LysoTracker intensities were determined using ImageJ (NIH). For estimation of lysosome number, we determined the number of LysoTracker-stained vesicles that were at least 0.1 μm^3^ in volume and divided the total number of vesicles per field with the number of cells in that field.

### Analysis of RNAi-Mediated Gene Knockdown.

Flies expressing the relevant RNAi transgenes or *UAS*-*Luc* under the control of heat shock–inducible promoter (hs-GAL4) were heat shocked in a 37 °C water bath for 1 h on 3 alternate days. The day after the third heat shock, RNA was extracted from whole-fly extracts using RNeasy mini kit (Qiagen) by following the manufacturer’s instructions. Using the high-capacity cDNA reverse transcription kit (Applied Biosystems), 1 μg of total RNA was reverse-transcribed. Real-time qPCR was performed using SYBR Green JumpStart Taq ReadyMix (Sigma) by following the manufacturer’s instructions. The primers used were as follows:

*rp49* (control):
F: 5′-CTA​AGC​TGT​CGC​ACA​AAT​GG-3′
R: 5′-GTT​GTG​CAC​CAG​GAA​CTT​CT-3′
itpr:
F: 5′-CTT​AAT​CCT​GAA​ATG​CAT​GTC​GG-3′
R: 5′-GGG​TAT​CTC​GCT​CCA​AAG​G-3′
trpml.:
F: 5′-TGA​CGG​CCG​ACT​GGA​ATT​C-3′
R: 5′-GGT​ATC​CCA​TTG​GTC​CAC​C-3′
mAchR:
F: 5′-CAA​ACA​GCA​GTG​ACG​AAA​ACA​C-3′
R: 5′-CAT​GTA​GAC​ACT​CTC​CGC​GT-3′
MCU:
F: 5′-CCA​CTG​GAA​GAG​AAA​AAA​CTG​G-3′
R: 5′-ATC​CCA​AGA​GTA​TTC​CCA​CCA-3′

### Statistical Analyses.

We used either a parametric or a nonparametric test of statistical significance on the basis of whether the data were normally distributed. Multiple comparisons were made by ANOVA. R, Excel (Microsoft), and Prism 8 (GraphPad) were used for statistical analyses. Custom R code used for quantifying data are available on GitHub (https://github.com/kvenkatachalam-lab/Wong-and-Karagas-et-al.-2021-PNAS-paper). Statistical significance was defined as a *P <* 0.05. *P* values were shown on the figures as asterisks: *P < 0.05; **P < 0.01; ***P < 0.001; and ****P < 0.0001. Lifespan (Kaplan–Meier) curves were generated using Prism 8. We used the log-rank (Mantel–Cox) test to determine *P* values.

## Supplementary Material

Supplementary File

## Data Availability

All study data are included in the article and/or supporting information. Custom R code used for quantifying data are available on GitHub (https://github.com/kvenkatachalam-lab/Wong-and-Karagas-et-al.-2021-PNAS-paper) (62).
