# Supplementary material for: Regulation of longevity by depolarization-induced activation of PLC-β–IP3R signaling in neurons
Source: Proc Natl Acad Sci U S A. 2021 Apr 15;118(16):e2004253118. doi: 10.1073/pnas.2004253118 (PMC8072327; doi:10.1073/pnas.2004253118)
Supplement: Supplementary File [file pnas.2004253118.sapp.pdf]

PNAS
www.pnas.org

Supplementary Materials for

Regulation of Lifespan by Depolarization-Induced Activation of PLCβ–IP_3R Signaling in Neurons

Ching-On Wong, Nicholas E. Karagas, Jewon Jung, Qiaochu Wang, Morgan A. Rousseau, Yufang Chao, Ryan Insolera, Pushpanjali Soppina, Catherine A. Collins, Yong Zhou, John F. Hancock, Michael X. Zhu, and Kartik Venkatachalam

Correspondence to: kartik.venkatachalam@uth.tmc.edu

This PDF file includes:

Figures S1 to S6

Legends for Figures S1 to S6

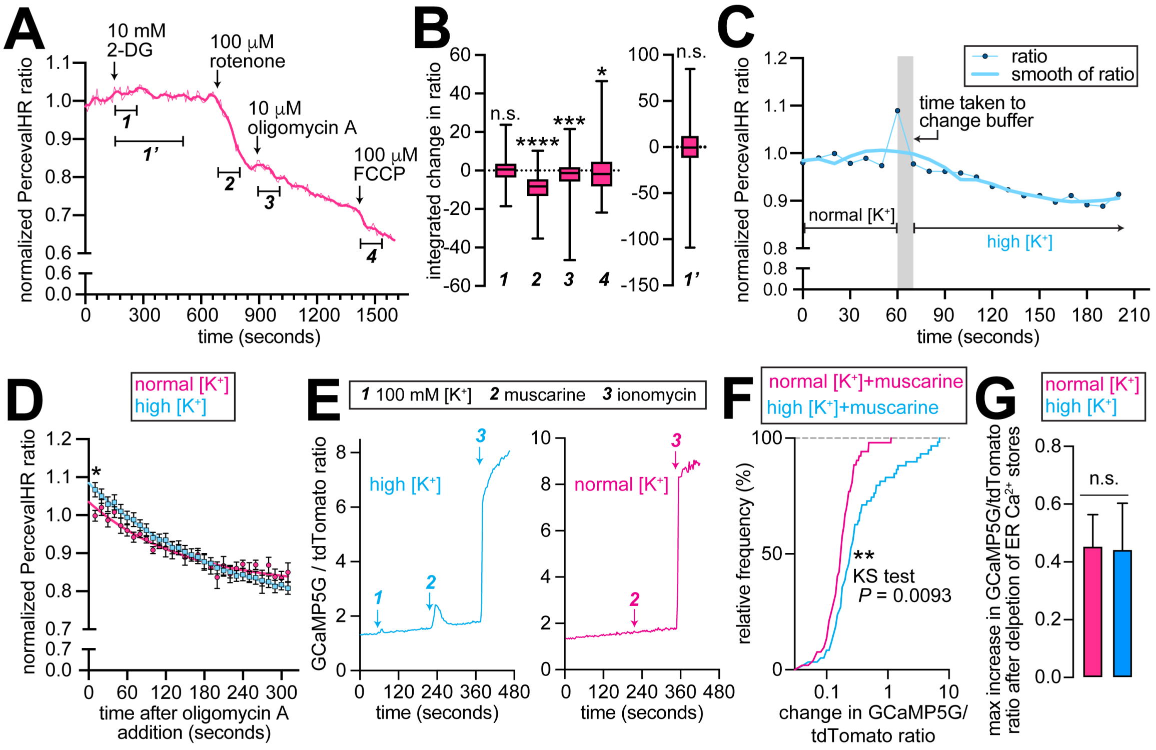

Fig. S1 – (**A**) Representative trace showing normalized PercevalHR ratio in *Drosophila* glutamatergic neurons. Arrows indicate treatments. ***1-4*** represented 2-minute windows after the addition of drug and ***1'*** represented a 6-minute window after 2-DG application. (**B**) Bar graphs showing the integrated change in PercevalHR ratio (relative to the starting values) over the durations indicated in (**A**). (**C**) PercevalHR ratio from Figure 1B (time: 0-200 seconds) showing that addition of 51.7 mM [K^+] did not result in a change in the PercevalHR ratio. *Grey box* represents the period during which the buffer containing 5 mM [K^+] was replaced with that containing 51.7 mM [K^+]. (**D**) Normalized PercevalHR ratio after oligomycin A treatment in depolarized (51.7 mM [K^+], *blue*) and normally polarized (5 mM [K^+], *pink*) neurons. Values represent mean ± SEM. *, P<0.05, t-test. Except for the 1st time point, none of the other time points showed significant differences in PercevalHR ratio between depolarized and polarized cells. (**E**)

Representative traces showing normalized GCaMP5G/tdTomato ratio in depolarized (51.7 mM [K^+], *blue*) and polarized (5 mM [K^+], *pink*) neurons. Arrows indicate treatments. (**F**) Cumulative distribution of muscarine-induced fold-change of GCaMP5G/tdTomato ratio from baseline values in depolarized (51.7 mM [K^+], *blue*) and polarized (5 mM [K^+], *pink*) neurons. **, $P < 0.01$, Kolmogorov-Smirnov test (KS test). (**G**) Bar graph showing maximum amplitude of changes in GCaMP5G/tdTomato ratio induced by thapsigargin (5 μM) in depolarized (51.7 mM [K^+], *blue*) and polarized (5 mM [K^+], *pink*) fly neurons in the absence of bath Ca^{2+}. Since thapsigargin depletes ER Ca^{2+} by blocking SERCA, increase in cytosolic [Ca^{2+}] (i.e., GCaMP5G/tdTomato ratio) reveals total ER Ca^{2+} content. Data represent median and 95% confidence intervals; n.s., not significant, Mann-Whitney test.

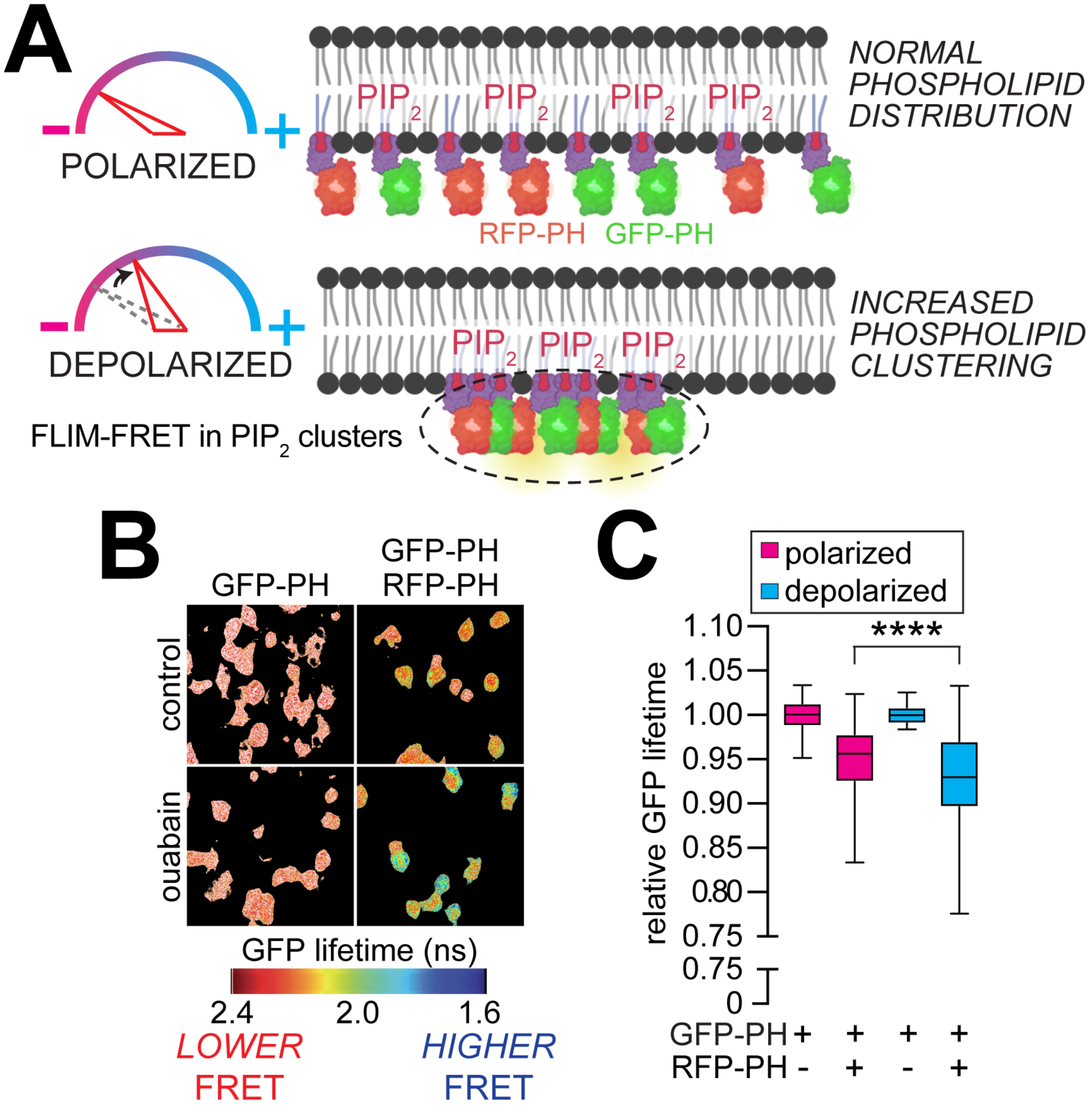

Fig. S2 – (**A**) PIP_2 molecules are bound by GFP-PH or RFP-PH. Plasma membrane depolarization causes clustering of PIP_2 and probes, which can be detected by FLIM-FRET. Image was created with BioRender.com. (**B**) Representative heat maps showing GFP lifetime in N2a cells expressing the indicated fluorophores under control or ouabain-treated conditions. (**C**) Boxplot showing quantification of relative GFP lifetime in control (*pink*) and ouabain-

treated (*blue*) cells. Values are normalized to the mean of GFP-PH-only datasets.

****, P<0.0001, t-test.

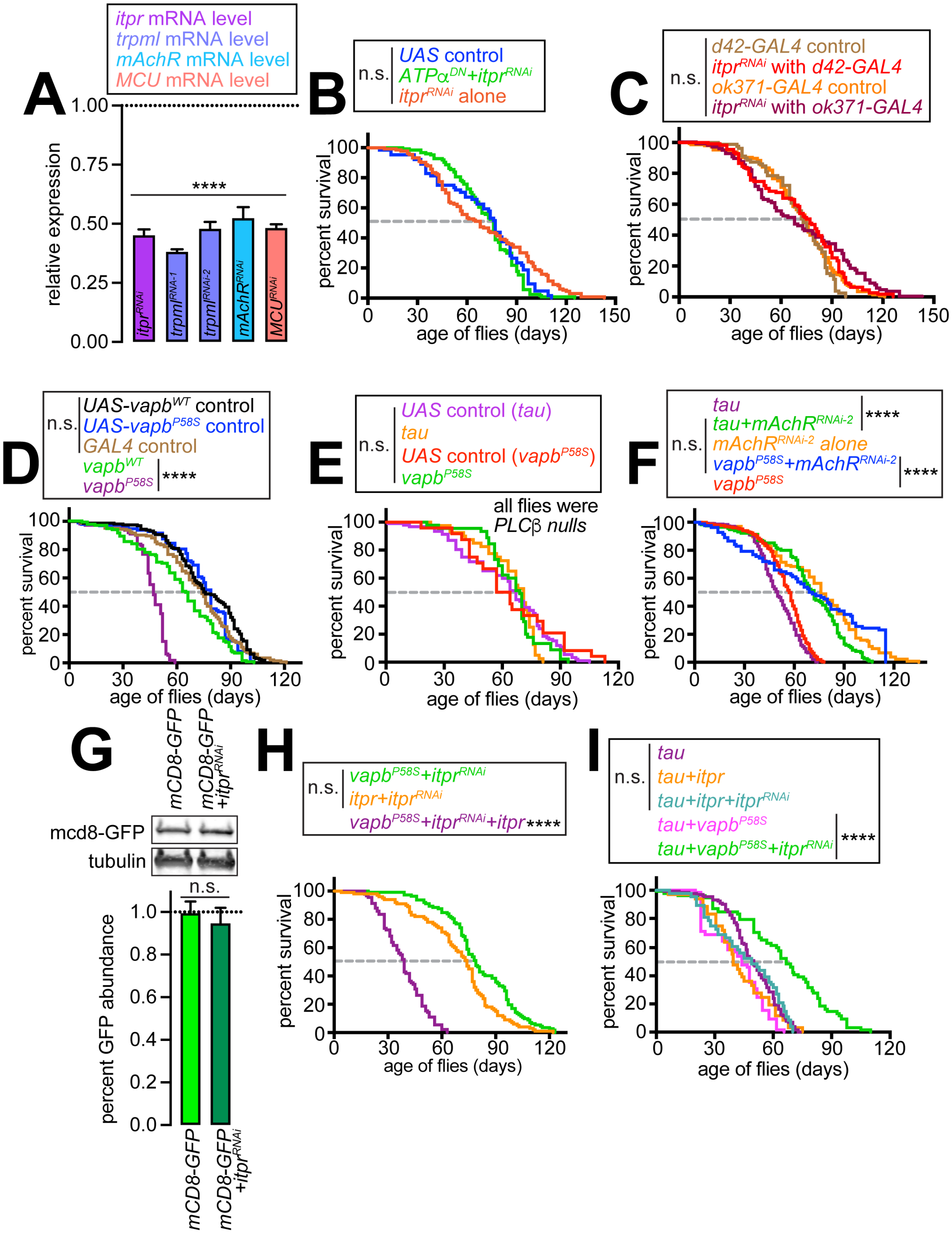

Fig. S3 – (**A**) Bar graph showing the relative mRNA levels of the indicated genes upon RNAi-mediated knockdown. Values were normalized to expression of gene in animals expressing luciferase (*hs>luciferase*) instead of the RNAi lines, and

represent mean ± SEM. All values were significantly different from controls. ****, *P*<0.0001, pairwise t-tests followed by Bonferroni corrections. (**B-F** and **H-I**) Lifespan of flies of the indicated genotypes. ****, *P*<0.0001, n.s., not significant, log-rank tests with Bonferroni correction. (**G**) *Top*, representative Western blot performed on fly head extracts generated from animals expressing the indicated transgenes in glutamatergic neurons. Blots were probed with antibodies to GFP and tubulin in flies. *Bottom*, bar graph depicting quantification of relative GFP abundance in the indicated genotypes. Bar graphs represent mean ± SEM. n.s., not significant, t-test.

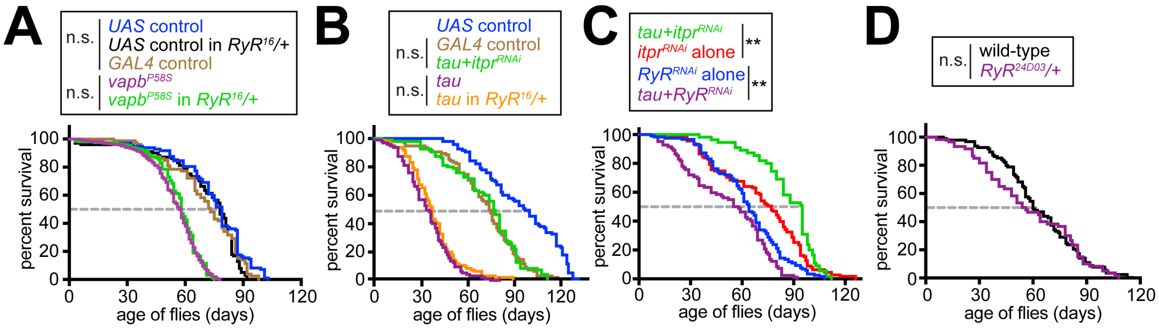

Fig. S4 – (**A-D**) Lifespan of flies of the indicated genotypes. *RyR*^{16} and *RyR*^{24D03} are null and genomic duplication alleles, respectively. **, *P*<0.005; n.s., not significant, log-rank tests with Bonferroni correction.

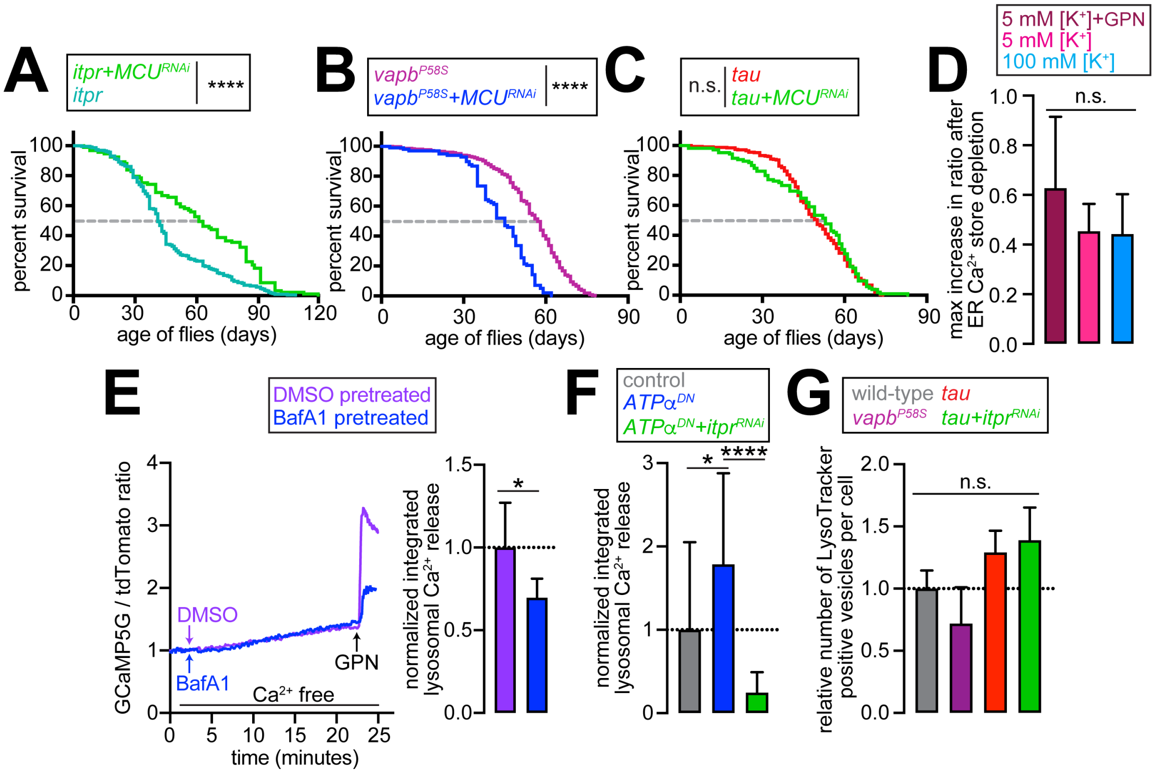

Fig. S5 – (**A-C**) Lifespan of flies of the indicated genotypes. ****, *P*<0.0001, n.s., not significant, log-rank tests with Bonferroni correction. (**D**) Bar graph showing maximum amplitude of thapsigargin-induced changes in GCaMP5G/tdTomato ratio in depolarized (51.7 mM [K$^+$], *blue*) and polarized (5 mM [K$^+$], *pink* and *maroon*) fly neurons in the absence of bath Ca^{2+}. In the GPN-pretreatment group, GPN (500 µM) was applied for 3.5 minutes prior to thapsigargin. Values represent median and 95% confidence intervals. n.s., not significant, Kruskal-Wallis test. (**E**) *Left*, representative traces showing GCaMP5G/tdTomato ratio in dissociated fly glutamatergic neurons. Arrows indicate points of drug application. *Right*, bar graphs quantifying GPN-induced changes in GCaMP5G/tdTomato ratio. Data represent median and 95% confidence intervals. *, *P*<0.05, Mann-Whitney test. (**F**) Bar graph quantifying GPN-induced changes in

GCaMP5G/tdTomato ratio in dissociated fly glutamatergic neurons expressing the indicated transgenes. Data represent median and 95% confidence intervals. *, *P*<0.05, ****, *P*<0.0001, Mann-Whitney tests with Bonferroni corrections. (**G**) Bar graph showing the relative number of LysoTracker-positive vesicles in glutamatergic neurons expressing the indicated transgenes. Values were normalized to lysosome number in wild-type neurons and represent mean ± SEM. n.s., not significant, ANOVA.

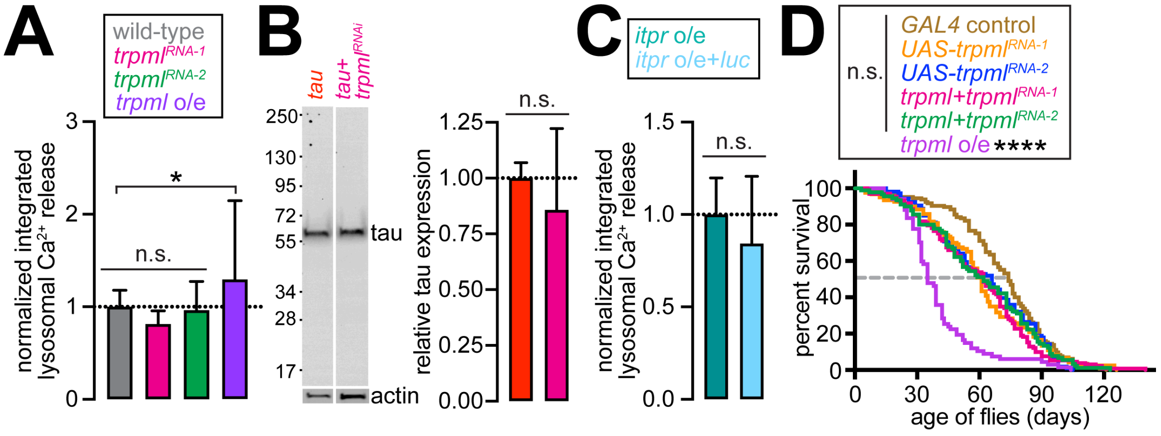

Fig. S6 – (**A** and **C**) Bar graphs quantifying GPN-induced changes in GCaMP5G/tdTomato ratio in dissociated fly glutamatergic neurons expressing the indicated transgenes. Data represent median and 95% confidence intervals. *, *P*<0.05, Mann-Whitney test, n.s., not significant, Kruskal-Wallis test (**A**) and Mann-Whitney test (**C**). (**B**) *Left*, representative Western blot showing larval brain extracts derived from animals of genotypes indicated on the top probed with antibodies against tau and actin. *Right*, bar graph showing quantification of the Western blot. Values represent mean ± SEM. n.s., not significant, t-test. (**D**) Lifespan of flies of the indicated genotypes. ****, *P*<0.0001, n.s., not significant, log-rank tests with Bonferroni correction.
